# Evaluation of the prothrombotic potential of four-factor prothrombin complex concentrate (4F-PCC) in animal models

**DOI:** 10.1371/journal.pone.0258192

**Published:** 2021-10-06

**Authors:** Subhajit Ghosh, Wilfred Krege, Baerbel Doerr, Marcel Mischnik, Ingo Pragst, Gerhard Dickneite, Eva Herzog

**Affiliations:** 1 Department of Pharmacology and Toxicology, CSL Behring GmbH, Marburg, Germany; 2 Department of Clinical Research and Development, CSL Behring GmbH, Marburg, Germany; University of Maryland Baltimore County, UNITED STATES

## Abstract

**Objectives:**

Acquired coagulopathy may be associated with bleeding risk. Approaches to restore haemostasis include administration of coagulation factor concentrates, but there are concerns regarding potential prothrombotic risk. The present study assessed the prothrombotic potential of four-factor prothrombin complex concentrate (4F-PCC) versus activated PCC (aPCC) and recombinant factor VIIa (rFVIIa), using three preclinical animal models.

**Methods:**

The first model was a modified Wessler model of venous stasis-induced thrombosis in rabbit, focusing on dilutional coagulopathy; the second model employed the same system but focused on direct oral anticoagulant reversal (i.e. edoxaban). The third model assessed the prothrombotic impact of 4F-PCC, aPCC and rFVIIa in a rat model of ferric chloride-induced arterial thrombosis.

**Results:**

In the first model, thrombi were observed at aPCC doses ≥10 IU/kg (therapeutic dose 100 IU/kg) and rFVIIa doses ≥50 μg/kg (therapeutic dose 90 μg/kg), but not 4F-PCC 50 IU/kg (therapeutic dose 50 IU/kg). The impact of 4F-PCC (up to 300 IU/kg) on thrombus formation was evident from 10 minutes post-administration, but not at 24 hours post-administration; this did not change with addition of tranexamic acid and/or fibrinogen concentrate. 4F-PCC-induced thrombus formation was lower after haemodilution versus non-haemodilution. In the second model, no prothrombotic effect was confirmed at 4F-PCC 50 IU/kg. The third model showed lower incidence of thrombus formation for 4F-PCC 50 IU/kg versus aPCC (50 U/kg) and rFVIIa (90 μg/kg).

**Conclusions:**

These results suggest that 4F-PCC has a low thrombotic potential versus aPCC or rFVIIa, supporting the clinical use of 4F-PCC for the treatment of coagulopathy-mediated bleeding.

## Introduction

A coagulopathic state may result from systemic disorders causing a reduction in coagulation factors (e.g. trauma). In addition, the coagulation response is modulated in patients who are in receipt of anticoagulation therapy (e.g. vitamin K antagonists [VKAs] or direct oral anticoagulants [DOACs]) at a level sufficient to prevent a thrombotic event. In both cases, there is an associated bleeding risk [1,2]. Techniques for haemostatic management of bleeding include controlling blood loss and replenishment of coagulation factors to prevent or ameliorate coagulopathy, and anticoagulant reversal (if applicable) [2,3].

It should be noted that coagulation factors and other haemostatic agents are often used sequentially or in combination in clinical practice [2,4]. Fresh frozen plasma (in conjunction with red blood cells) is one recommendation for the initial management of patients with massive traumatic haemorrhage [2], but it is not routinely recommended in guidelines on anticoagulation reversal, partly due to the fact that its clotting factor content is lower than typical levels in whole blood [5,6]. As such, factor concentrates, with their greater concentrations of clotting factors within a lower administration volume [7,8], have been included in treatment protocols, with some studies indicating similar, or improvements, in patient outcomes versus fresh frozen plasma alone [4,9–11]. Fibrinogen is known to be depleted in trauma [12], and so early administration of fibrinogen concentrate (FCH) is recommended by the Task Force for Advanced Bleeding Care in Trauma [2]; maintenance of a target fibrinogen level is also recommended by the American College of Cardiology when managing DOAC-associated bleeding [3]. Tranexamic acid (TXA) is a further option, with early administration recommended in the initial management of bleeding and coagulopathy [2,3].

Prothrombin complex concentrates (PCCs) can be used to treat trauma-induced bleeding (e.g. where a deficit in clotting factors may be present) and are also frequently used for anticoagulation reversal. PCCs consist of preparations of activated or non-activated vitamin K-dependent coagulation factors (F); non-activated PCCs include 4F-PCCs (containing FII, VII, IX and X) and 3F-PCCs (containing only minimal amounts of FVII alongside FII, IX and X) [13], while activated PCC (aPCC) includes mainly non-activated FII, IX and X, in conjunction with mainly activated FVII [14]. Recombinant activated FVII (rFVIIa) is also available as a stand-alone product, but is not recommended to treat trauma unless all other attempts to control bleeding have failed [2]; it is noted as a treatment option for anticoagulation reversal in some guidelines, but is not routinely recommended as a first-line option [6,15–17].

As with other procoagulants, 4F-PCC use carries a potential risk of “over-correction” of coagulopathy, potentially leading to thromboembolic events (TEE). To date, clinical data available on the safety profile of 4F-PCC in trauma are limited. 4F-PCC is an established clinical treatment for VKA reversal, with TEE risk similar to that of plasma [10,11,18]; data on the thrombotic safety of 4F-PCC in DOAC reversal (which lies outside the licensed indication for 4F-PCC) are more limited [19–22], although animal studies generally show a good safety profile [19–21]. Data from pig models of dilutional coagulopathy have suggested that PCC treatment is associated with improved outcomes (i.e. reduced time to haemostasis, augmented thrombin generation and higher survival rates versus comparators) [23–26], although individual reports suggest that there may be a risk of TEE at a 4F-PCC dose of 50 IU/kg [27].

To further investigate the thrombotic safety of 4F-PCC with respect to other haemostatic agents, we conducted a series of experiments in various animal models. In the first experiment, the thrombotic activities of 4F-PCC, aPCC and rFVIIa were investigated in a model of dilutional coagulopathy followed by induction of venous stasis-induced thrombosis in rabbits using the well-established Wessler model [19,28–31]. The same test agents were also administered to rabbits anticoagulated with the direct FXa inhibitor edoxaban so as to evaluate their potential thrombotic activities in the context of direct oral anticoagulation. Finally, in order to further assess their prothrombotic potential, 4F-PCC, aPCC and rFVIIa were administered to rats using a ferric chloride-induced arterial thrombosis model.

## Methods

All animals received care in compliance with the European Convention on Animal Care, and the studies were approved by local governmental authorities (Regierungspraesidium Gießen). The animal ethics approval numbers were V54-19 c 2015 c MR 1 Nr. A 21/2011 and V54-19 c 2015 c MR 1 Nr. 8a-04-35. All health monitoring was conducted by qualified and registered personnel. Animals were monitored at least 3 times daily during general husbandry and continuously during experiment conduct. For rats, anaesthesia was initiated by an intraperitoneal injection and maintained via intravenous (IV) infusion into the lateral tail vein of the ketamine/xylazine mixture. For rabbits, anaesthesia was induced with 5 mg/kg IV ketamine (Ketavet^®^, Pharmacia GmbH, Erlangen, Germany) and 0.5 mg/kg IV xylazine 2% (Rompun^®^, Bayer Vital GmbH, Leverkusen, Germany) and maintained with isoflurane (Isofluran CP^®^, CP-Pharma Handelsgesellschaft GmbH, Burgdorf, Germany). The method of euthanasia employed was an overdose of CO_2_ or Isofluran for rats and barbiturates (Euthadorm^®^) for rabbits, respectively.

The severity of all procedures applied as part of this study were categorised to be of low grade as all procedures were conducted under full anaesthesia and therefore warranted to be pain free. In addition, all procedures including anaesthesia were terminal. No animals were terminated prior to the conclusion of the procedure. Sample size was determined based on historical experience with the animal models applied including expected attrition rate, effect size, and data variability.

### Dilutional coagulopathy and venous stasis in rabbits

#### Animals

Female New Zealand White rabbits 3–4 months old and weighing 2.2–3.2 kg (Manfred Bauer, Neuenstein-Lohe, Germany) received care in compliance with the European Convention on Animal Care, and the study was approved by the local governmental authorities. The animals were housed individually in wire-steel cages at 21–23°C and 50% relative humidity under a 12 h/12 h light-darkness cycle. The animals had free access to tap water and were fed rabbit pellets (Deukanin, Deutsche Tiernahrung Cremer GmbH & Co. KG, Düsseldorf, Germany) ad libitum.

#### Venous stasis

Venous stasis was induced 10 minutes after the end of infusion; for analysis of longitudinal response to 4F-PCC, stasis was induced at varying intervals up to 168 hours post-infusion. Any observed thrombi were graded according to a scoring system from 0 to 3, and their wet weights were determined. Thrombus scores were defined as: 0 (no clot), 1 (one or a low number of small clots, too small to determine weights), 2 (not fully occluding clot, with measurable weight) or 3 (fully occluded clot). Histopathological investigations were also performed.

#### Modified Wessler test

**Fig 1:**
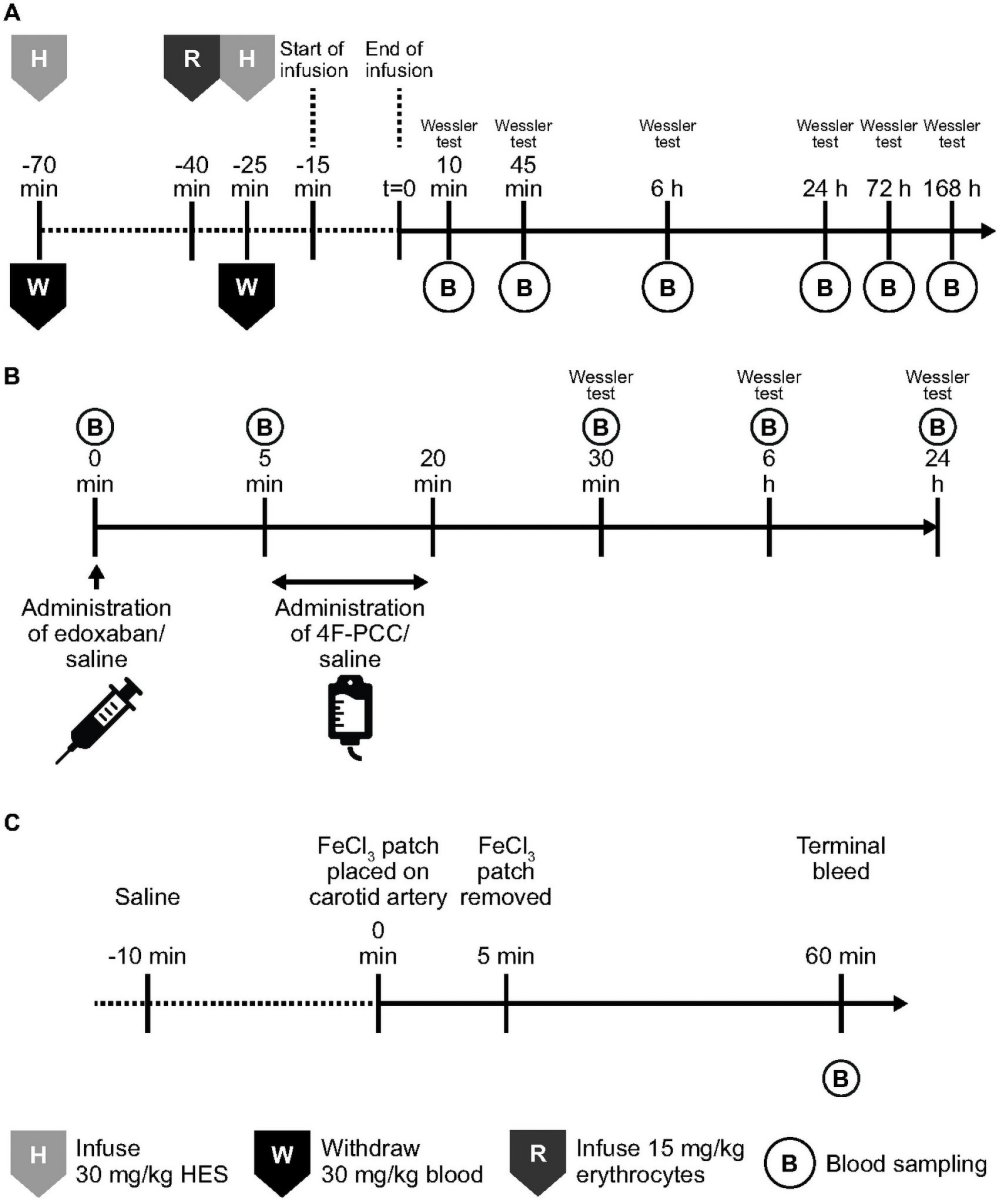
Study schematics. (A) 4F-PCC in rabbits with dilutional coagulopathy, using modified Wessler model of venous stasis-induced thrombosis; (B) 4F-PCC in rabbits with edoxaban-induced coagulopathy using modified Wessler model of venous stasis-induced thrombosis; (C) 4F-PCC in rats, using a ferric chloride-induced model of arterial thrombosis. Haemodilution was used only in the experiments indicated. 4F-PCC, 4-factor prothrombin complex concentrate; h, hours; min, minutes; t, time.

To assess thrombogenicity, we used a modification of the Wessler model of venous stasis-induced thrombosis in rabbits [28–30], as described previously [19] and illustrated in Fig 1. The primary endpoints of the study were thrombus score (based on number of clots, degree of occlusion and potential for determination of measurable thrombus weight) and thrombus wet weight after venous thrombosis. Secondary endpoints included assessment of histopathology.

For analysis of longitudinal response to 4F-PCC, venous stasis was induced at 10 minutes, 45 minutes, 6 hours, 24 hours, 72 hours and 168 hours post-infusion. Procoagulant effects were determined by exposing the contralateral jugular vein and isolating a segment of approximately 2 cm, causing a complete stasis in the isolated segment. Thirty minutes after stasis induction the vein segment was excised and dissected in sodium citrate solution.

#### Haemodilution and treatments

Female New Zealand White rabbits were subjected to two cycles of haemodilution, separated by a 45-minute interval; animals received 15 mL/kg salvaged erythrocytes part way through this interval (Fig 1A). Haemodilution was only used in some experiments; where haemodilution was used, it was performed in advance of any treatment administration. Treatments included 4F-PCC, aPCC, rFVIIa, TXA and FCH. Animals were randomly allocated to treatment groups (n = 5–18 per group). Treatments were administered via IV infusion, with doses selected to range from therapeutic to supratherapeutic. TXA (15 mg/kg) was administered 5 minutes prior to 4F-PCC infusion as an IV bolus, as was FCH (100 mg/kg); FCH administration continued in parallel with 4F-PCC administration.

Haemodilution was conducted as described in [1]. Animals were subjected to haemodilution in phases by withdrawal of 30 mL/kg blood and infusion of 30 mL/kg hydroxyethyl starch (HES; Infucoll^®^ 6% solution; Schwarz Pharma) pre-warmed to 37°C. This procedure was repeated after 45 minutes. After 30 minutes, during the interval between the two cycles of blood withdrawal and HES infusion, the animals received 15 mL/kg salvaged erythrocytes, prepared from withdrawn whole rabbit blood by centrifugation for 10 min at 800×g, washing in normal saline and resuspension in Ringer’s lactate.

4F-PCC (Beriplex^®^/Kcentra^®^, CSL Behring GmbH, Marburg, Germany) was reconstituted in water for injection (B. Braun Melsungen AG, Melsungen, Germany) to a final FIX potency of 28 IU/mL, as per label instructions [2], and administered via IV infusion at doses of 50, 100, 200, 300, 400 and 500 IU/kg. 3F-PCC (Bebulin^®^ VH, Baxter Healthcare Corporation, Westlake Village, USA) [3] was reconstituted in sterile water for injection and administered at 300 IU/kg. Activated PCC (Factor Eight Bypassing Activity [FEIBA] Baxter AG, Vienna, Austria) [4] was reconstituted in water for injection and administered at doses of 5, 10, 50, 75 and 100 IU/kg. rFVIIa (NovoSeven^®^, Novo Nordisk A/S, Bagsværd, Denmark) [5] was reconstituted in histidine diluent and administered at doses of 10, 50, 90, 180 and 300 μg/kg.

TXA (Carinopharm GmbH, Elze, Germany; provided as solution [100 mg/mL]) was administered at a dose level of 15 mg/kg, 5 minutes prior to 4F-PCC infusion as an IV bolus; this was considered a therapeutic dose. FCH (RiaSTAP^®^, CSL Behring GmbH, Marburg, Germany) was reconstituted in water for injection and a dose of 100 mg/kg was administered as an IV bolus beginning 5 minutes prior to 4F-PCC infusion and continuing in parallel with 4F-PCC administration [6]. Equivalent volumes of isotonic saline [0.9%, Fresenius Kabi Deutschland GmbH, Bad Homburg, Germany] were used in the negative control groups. In rabbits with dilutional coagulopathy, there were 29 groups in total with n = 5–18 per group.

#### Histopathology

Following necropsy, the lung, kidney, liver, heart and brain were harvested and fixed in 10% Neutral Buffered Formalin for histopathological analysis. Histopathological investigations (Covance Laboratories, Harrogate, UK) of lung, kidney, liver, heart and brain tissue were conducted for all treatment groups for positive signals of thrombus formation following venous stasis. Tissue was processed and embedded in paraffin wax blocks, and sections with a nominal thickness of 5 μm were stained with haematoxylin and eosin prior to examination. Histopathology was assessed in accordance with the agreed definitive Histopathology Technical Method.

### Direct FXa-induced coagulopathy in rabbits

To further evaluate the thrombogenicity of 4F-PCC, we investigated its impact in rabbits undergoing edoxaban anticoagulation, using the modified Wessler model as described above. Specifics of the animals used for these experiments are as above for dilutional coagulopathy, as are details of 4F-PCC, aPCC and rFVIIa preparation, and histopathology.

#### Study design

The study design is illustrated in Fig 1B. The primary endpoints were thrombus score in the presence and absence of edoxaban, thrombus wet weight after venous thrombosis and histopathological changes in lungs, kidney, liver, heart and brain tissue.

#### Treatments and model

Animals were randomly allocated to treatment groups (n = 5 per group). Edoxaban tosilate hydrate (600 μg/kg) was administered via slow IV bolus at 0 minutes (Fig 1B). The dose level was chosen to reflect therapeutically relevant doses of edoxaban in this model, avoiding excessive anticoagulation [32].

4F-PCC doses of 50 and 300 IU/kg were administered via IV infusion over 15 minutes, starting at 5 minutes (Fig 1B); doses were chosen to include the 4F-PCC dose required to reverse edoxaban-associated bleeding in previous experiments in a rabbit model (i.e. 50 IU/kg) [32], and supra-therapeutic doses expected to elicit a reproducible prothrombotic response in this animal model (i.e. 300 IU/kg) [31].

Edoxaban tosilate hydrate powder (Lixiana^®^, Daiichi Sankyo Co., Ltd, Tokyo, Japan) was reconstituted in Glucosteril^®^ 5% (Fresenius Kabi Deutschland GmbH, Bad Homburg, Germany) to a final concentration of 300 or 600 μg/mL [7].

4F-PCC (Beriplex^®^/Kcentra^®^, CSL Behring GmbH, Marburg, Germany) was prepared as described in the section above, and was administered via the ear vein. Equivalent volumes of isotonic saline (0.9%, Fresenius Kabi Deutschland GmbH, Bad Homburg, Germany) were used as negative control. There were 10 experimental groups with n = 5 per group.

### Arterial thrombosis in rats

#### Study design

The thrombogenicity of 4F-PCC in comparison with aPCC and rFVIIa was assessed using a ferric chloride (FeCl_3_)-induced arterial thrombosis model in rats. Primary endpoint was the incidence of thrombus occlusion and time to occlusion.

#### Animals

Female CD (Sprague Dawley wildtype) IGS rats 9–12 weeks old and weighing 230–260 g (Charles River, Neuenstein-Lohe, Germany) received care in compliance with the European Convention on Animal Care, and the study was approved by the local governmental authorities. The animals were housed in macrolon cages with wood shavings (J Rettenmaier und Söhne, Rosenberg, Germany) at 20–24°C and 40–50% relative humidity under a 12 h/12 h light-darkness cycle. The animals had free access to tap water and were fed a standard rat diet (Ssniff-Versuchsdiäten, Soest, Germany). Rats were housed under laboratory conditions, after a health examination. Only animals who passed this examination and had no visible signs of illness were used for the study.

#### Treatment and arterial thrombosis model

A 1 x 2.5 mm filter paper patch saturated with FeCl_3_ solution was placed on the carotid artery for a 5-minute period in order to induce thrombosis; varying concentrations of FeCl_3_ (5%, 7.5%, 10%, and 50% solutions) were initially applied to establish the optimum concentration for use in this study.

4F-PCC was given by intravenous infusion, while aPCC, rFVIIa and saline were administered as a slow IV bolus injection via the jugular vein; infusion time points were 10 minutes prior to thrombus induction for aPCC, and 5 minutes prior to thrombus induction for 4F-PCC and rFVIIa. Isotonic saline (2 mL/kg; 0.9%, Fresenius Kabi Deutschland GmbH, Bad Homburg, Germany) served as the negative control.

When administering triple treatments, a total of 2.24 mL injection volume was used across all groups; part of this volume was the group’s treatment product, while the remaining volume was substituted using isotonic saline. Considering the FeCl_3_ treatment as t = 0, the three treatments were given at -5, -10 and -15 minutes; for 4F-PCC, isotonic saline was given at -15 and -10 min while 4F-PCC was given at -5 min, while for isotonic saline triple, saline was given at -15, -10 and -5 min. Blood flow was monitored for 60 minutes following FeCl_3_ patch application, after which blood samples were terminally drawn through puncturing the vena cava. Blood flow in rats was measured with the TS420 Perivascular flow module and using 1.5PSL flow probes (Transonic).

Thrombosis was induced via placement of a filter paper patch saturated with FeCl_3_ solution on the carotid artery; varying concentrations of FeCl_3_ were applied in order to induce a sub-maximal incidence of thrombus formation, thus allowing observation of potential pro-coagulatory effects. Both the 5% and 7.5% concentrations of FeCl_3_ solution achieved a thrombus incidence rate below 100% (20% and 70% for 5% FeCl_3_ and 7.5% FeCl_3_, respectively); therefore the 5% FeCl_3_ concentration was chosen for investigation with the agents, as mostly procoagulant effects were expected. Blood flow was monitored for 60 minutes (Fig 1C) and terminal blood samples were subsequently drawn. 4F-PCC, aPCC or rFVIIa were administered as an IV bolus. There were 7 experimental groups with n = 10 per group.

### Statistical analysis

As indicated in the results below, groups were pooled for analysis. Quantitative variables were summarised using the mean and standard deviation/standard error. P-values were calculated by Wilcoxon rank sum-test. Power analysis was done against the t-distribution with a significance level of 0.05. Randomisation of animals to study groups was not used in most experiments. Operators were not blinded during the study. No methods were used to reduce subjective bias.

## Results

### Dilutional coagulopathy in rabbits: Impact of 4F-PCC, aPCC and rFVIIa

#### Dose-response for 4F-PCC, aPCC, and rFVIIa

**Fig 2:**
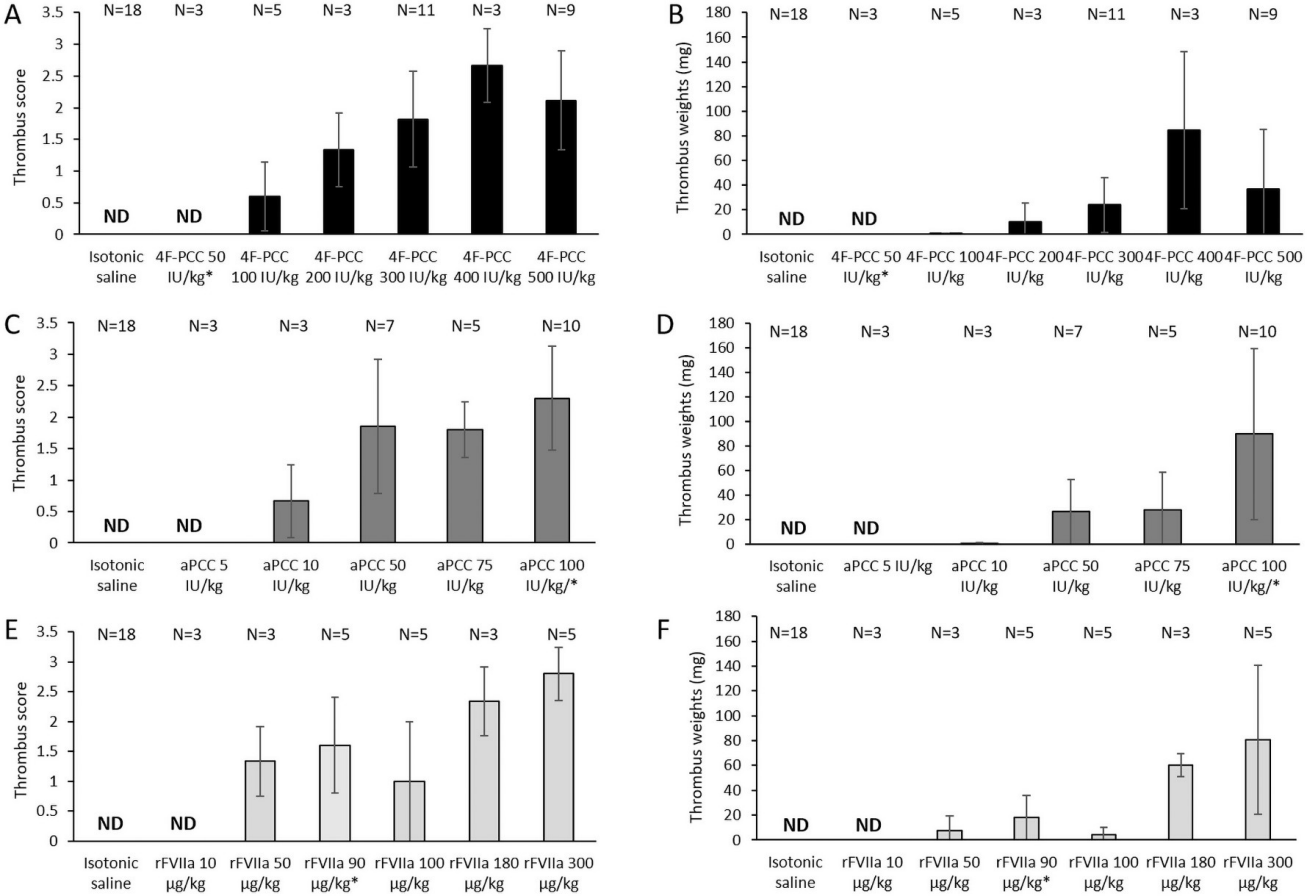
Thrombus score and thrombus weight: Dose-response for 4F-PCC, aPCC, and rFVIIa. (A) 4F-PCC thrombus score; (B) 4F-PCC thrombus weight; (C) aPCC thrombus score; (D) aPCC thrombus weight; (E) rFVIIa thrombus score; (F) rFVIIa thrombus weight. Data for venous stasis-induced thrombosis 10 minutes after the end of PCC, aPCC or rFVIIa infusion. Figure shows mean (standard deviation). * indicates a therapeutic dose.

At the lower end of the dose ranges tested (4F-PCC 50 IU/kg, aPCC 5 IU/kg, rFVIIa 10 μg/kg) we did not detect any thrombus formation when venous stasis was induced (10 minutes after infusion ended; Fig 2). For 4F-PCC, thrombus formation started to be observed at doses of 100 IU/kg or above (Fig 2A); however, thrombus weights were below 50 mg until doses exceeded 400 IU/kg (Fig 2B). At these higher doses, thrombus weight was correlated with thrombus score. At 4F-PCC 500 IU/kg, mean thrombus score was 2.1 (standard deviation [SD] 0.78) and mean thrombus weight was 37.11 mg (SD 48.37). For aPCC, thrombi were observed at doses of 10 IU/kg or above, with thrombus scores of approximately 2 for doses of 50 IU/kg or above (Fig 2C). Thrombus weight was 26.29 mg (SD 26.37) for the 50 IU/kg dose and increased further with increasing dose (Fig 2D). With rFVIIa, thrombi were observed at doses of 50 μg/kg (score 1.33 [SD 0.58]) and above (Fig 2E). Thrombus weights were under 10 mg at rFVIIa 50 μg/kg (7.68 mg [SD 11.55]) but increased to 60.33 mg (SD 9.07) at the 180 μg/kg dose (Fig 2F). At therapeutic doses (i.e. 4F-PCC 50 IU/kg, rFVIIa 90 μg/kg and aPCC 100 IU/kg), we did not observe thrombi with 4F-PCC but did for rFVIIa and aPCC (mean [SD] thrombus scores of 1.60 [0.89] and 2.30 [0.82] respectively). Reflecting thrombus scores of the treatments tested, aPCC was associated with the greatest mean thrombus weight: 89.80 mg (SD 69.82) at 100 IU/kg.

#### Time course of response to 4F-PCC

**Fig 3:**
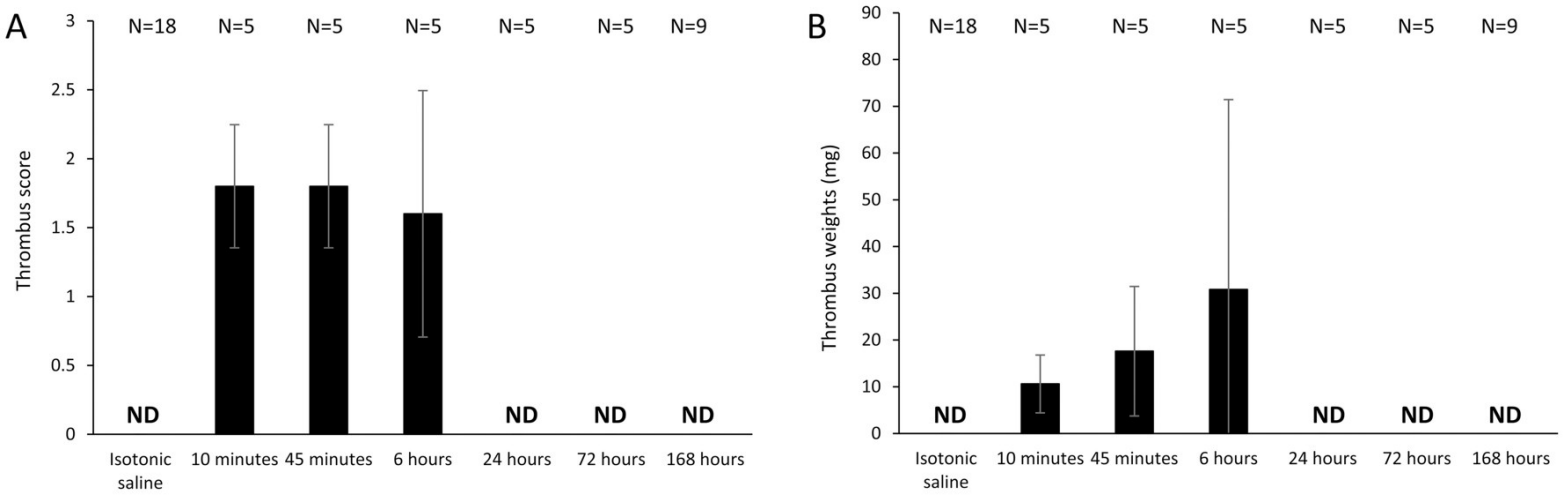
Time course of response to 4F-PCC. (A) thrombus score; (B) thrombus weight. ND, not detectable. Data for venous stasis-induced thrombosis at the indicated timepoints after the end of 4F-PCC (300 IU/kg) infusion. Figure shows mean (standard deviation).

The thrombus formation seen at the supratherapeutic dose of 300 IU/kg of 4F-PCC at 10 minutes post-administration (approximate score: 2) was also confirmed at 45 minutes and 6 hours post dosing (Fig 3A). The effects of 4F-PCC were no longer apparent at 24 hours; testing carried out at 1 week (168 hours) post-administration gave a thrombus score of 0. Thrombus weights were generally correlated with thrombus scores (Fig 3B); mean weights at 10 minutes and at 45 minutes were 10.60 mg (SD 6.19) and 17.60 mg (SD 13.85), respectively. Although we noted a further increase in thrombus weight from the 45-minute to the 6-hour time point, the large error (SD) observed at 6 hours does not allow any firm conclusions to be drawn.

#### 4F-PCC in combination with TXA and/or FCH

Thrombus scores following a supratherapeutic dose of 4F-PCC (300 IU/kg: 1.82 [SD 0.75]) were in the same range as scores for 4F-PCC in combination with TXA 15 mg/kg (2.00 [SD 0.00]), 4F-PCC in combination with FCH 100 mg/kg (1.60 [SD 0.55]), and 4F-PCC in combination with both TXA, and FCH (0.80 [SD 1.10]; S1 Fig). TXA 15 mg/kg or FCH alone did not lead to detectable thrombus formation. Thrombus weights were as follows: 4F-PCC alone (300 IU/kg: 9.0 mg [SD 9.82]), 4F-PCC with TXA 15 mg/kg (18.0 mg [SD 12.63]), 4F-PCC with FCH 100 mg/kg (4.8 mg [SD 6.38]), 4F-PCC with TXA and FCH (13.4 [SD 28.32]).

#### 4F-PCC in haemodilution

In animals who had undergone haemodilution (Table 1), thrombus scores and weights with a supratherapeutic dose of 4F-PCC (300 IU/kg: thrombus score 1.40 [SD 0.89], thrombus weight 9.0 mg [SD 9.8]) were similar regardless of whether animals also received 100 mg/kg FCH (thrombus score 0.80 [1.10], thrombus weight 11.4 mg [SD 17.9]; S2 Fig). Thrombus scores and weights were lower in haemodiluted animals compared with non-haemodiluted animals, both in terms of thrombus score (1.4 [SD 0.89] vs 1.8 [SD 0.8] respectively) and weight (9.0 mg [SD 9.8] vs 23.9 mg [SD 22.4] respectively).

**Table 1:** Study treatment groups. (A) 4F-PCC in rabbits with dilutional coagulopathy, using modified Wessler model of venous stasis-induced thrombosis; (B) 4F-PCC in rabbits with edoxaban-induced coagulopathy using modified Wessler model of venous stasis-induced thrombosis; (C) 4F-PCC in rats, using a ferric chloride-induced model of arterial thrombosis.

**A**					
**Group** *	**Treatment (dose)**	**Additional treatment**	**Venous stasis-induced (minutes/hours after last treatment)**	**Results reported in conjunction with group(s)**	**Year of study**
1	Isotonic saline	-	10 minutes		2003, 2008, 2009, 2013
2	4F-PCC (50 IU/kg)	-	10 minutes	1, 12, 15	2009
3	4F-PCC (100 IU/kg)	-	10 minutes	1	2008
4	4F-PCC (200 IU/kg)	-	10 minutes	1	2009
5	4F-PCC (300 IU/kg)^†^		10 minutes	1	2009, 2015
6	4F-PCC (400 IU/kg)	-	10 minutes	1	2009
7	4F-PCC (500 IU/kg)^†^		10 minutes	1	2009, 2015
8	aPCC (5 IU/kg)	-	10 minutes	1	2009
9	aPCC (10 IU/kg)	-	10 minutes	1	2009
10	aPCC (50 IU/kg)	-	10 minutes	1	2003, 2012
11	aPCC (75 IU/kg)^†^		10 minutes	1	2015
12	aPCC (100 IU/kg)	-	10 minutes	1	2003, 2008
13	rFVIIa (10 μg/kg)	-	10 minutes	1	2009
14	rFVIIa (50 μg/kg)	-	10 minutes	1	2009
15	rFVIIa (90 μg/kg)	-	10 minutes	1	2016
16	rFVIIa (100 μg/kg)	-	10 minutes	1	2003
17	rFVIIa (180 μg/kg)	-	10 minutes	1	2009
18	rFVIIa (300 μg/kg)	-	10 minutes	1	2003
19	4F-PCC (300 IU/kg)	-	45 minutes	1, 5	2013
20	4F-PCC (300 IU/kg)	-	6 hours	1, 5	2013
21	4F-PCC (300 IU/kg)	-	24 hours	1, 5	2013
22	4F-PCC (300 IU/kg)	-	72 hours	1, 5	2013
23	4F-PCC (300 IU/kg)	-	168 hours	1, 5	2013
24	Isotonic saline	TXA	10 minutes		2015
25	4F-PCC (300 IU/kg)	TXA	10 minutes	24	2015
26	4F-PCC (300 IU/kg)^†^	FCH	10 minutes	24	2015
27	4F-PCC (300 IU/kg)	TXA, FCH	10 minutes	24	2015
28**	4F-PCC (300 IU/kg) ^†^	Saline	10 minutes	24	2015
29**	4F-PCC (300 IU/kg)^†^	FCH	10 minutes	28	2015

**B**				
Group*	**First treatment (dose) (t = 0 minutes)**	**Second treatment (t = 5–20 minutes)**	**Venous stasis-induced (minutes/hours after edoxaban dosing)**	**Year of study**
1	Isotonic saline (1.0 mL/kg)	Isotonic saline (10.7 mL/kg)	30 minutes	2014
2	Isotonic saline (1.0 mL/kg)	4F-PCC (50 IU/kg)	30 minutes	
3	Isotonic saline (1.0 mL/kg)	4F-PCC (300 IU/kg)	30 minutes	
4	Edoxaban (600 μg/kg)	Isotonic saline (10.7 mL/kg)	30 minutes	
5	Edoxaban (600 μg/kg)	4F-PCC (50 IU/kg)	30 minutes	
6	Edoxaban (600 μg/kg)	4F-PCC (300 IU/kg)	30 minutes	
7	Edoxaban (600 μg/kg)	4F-PCC (300 IU/kg)	6 hours	
8	Edoxaban (600 μg/kg)	4F-PCC (300 IU/kg)	24 hours	
9	Isotonic saline (1.0 mL/kg)	4F-PCC (300 IU/kg)	6 hours	
10	Isotonic saline (1.0 mL/kg)	4F-PCC (300 IU/kg)	24 hours	

**C**		
Group*	**Treatment**	**Year of study**
1	Isotonic Saline 0.9%	2016
2	Isotonic Saline (triple treatment)	
3	4F-PCC (50 IU/kg)	
4	4F-PCC (100 IU/kg)	
5	4F-PCC (150 IU/kg)	
6	aPCC (50 IU/kg)	
7	rFVIIa (90 μg/kg)	

A

4F, four-factor; FCH, fibrinogen concentrate 100 mg/kg; IU, international units; rFVIIa, recombinant factor VIIa; PCC, prothrombin complex concentrate; TXA, tranexamic acid 15 mg/kg.

*n = 5–18 per group.

**Only Groups 28 and 29 underwent haemodilution.

†Pretreated with isotonic saline.

B

4F, four-factor; IU, international unit; PCC, prothrombin complex concentrate.

*n = 5 per group.

C

4F, four-factor; IU, international units; PCC prothrombin complex concentrate.

*n = 10 for each.

FeCl_3_ concentration 5%.

#### Histopathology

No thrombus formation was observed in the kidney, liver, heart or brain. Thrombus formation was not found in the lung in animals receiving 4F-PCC (50 IU/kg). In the lung of animals treated with a supratherapeutic dose of 4F-PCC 300 IU/kg, thrombus formation was recorded, consistent with administration of a prothrombotic agent and the results of the Wessler rabbit model. There was no evidence of thrombus formation until 1.25 hours post-dose. At this timepoint we observed minimal lung thrombi formation (grade 1) in 2/5 animals and grade 3 thrombus formation in 1/5 animals. There was a general decline in thrombus incidence at and after 24.5 hours post-dose.

### Direct FXa inhibitor-induced coagulopathy in rabbits: Impact of 4F-PCC

#### Dose-response for 4F-PCC

**Fig 4:**
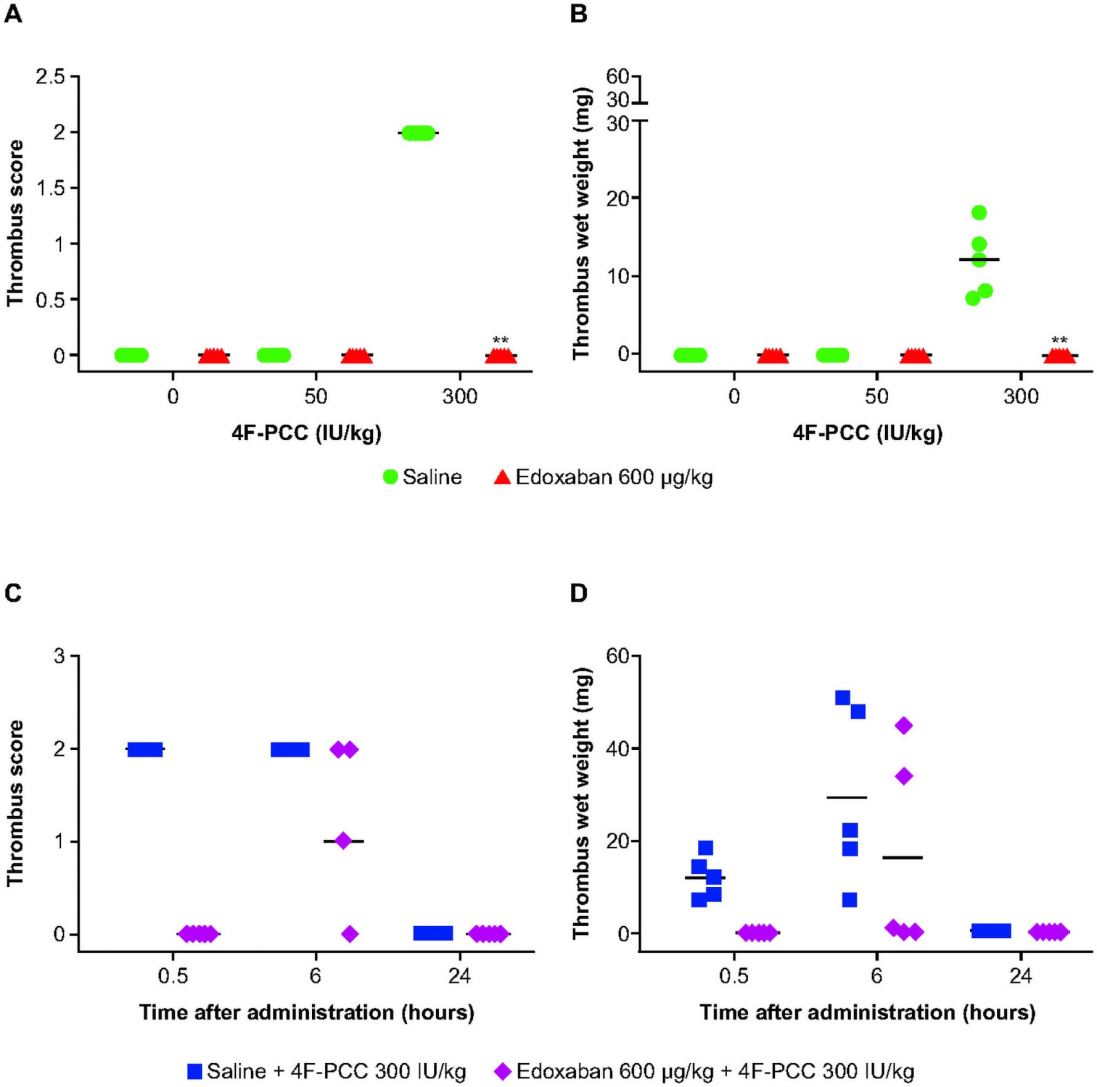
Data for venous stasis-induced thrombosis following end of PCC infusion. (A) Thrombus score and (B) thrombus wet weight at 30 minutes following administration of edoxaban (600 μg/kg) or saline followed by 4F-PCC and venous stasis in rabbits; (C) thrombus score and (D) thrombus wet weight over time following administration of edoxaban 600 μg/kg or saline followed by 4F-PCC (300 IU/kg) and venous stasis in rabbits. Figure shows median (range). **P<0.01.

Following administration of saline or 4F-PCC 50 IU/kg, no thrombus formation was observed at 30 minutes (Fig 4A and 4B). When the supratherapeutic dose of 4F-PCC 300 IU/kg was administered, thrombus formation with score 2 was observed in all animals at both 30 minutes and 6 hours, with median (range) thrombus wet weights of 12 (7–18) mg and 22 (7–51) mg, respectively (Fig 4C and 4D), confirming prior results. Thrombus formation was no longer evident at 24 hours (Fig 4C and 4D). Administration of a clinically relevant dose of edoxaban 600 μg/kg prior to 4F-PCC 300 IU/kg significantly inhibited thrombus formation 30 minutes after edoxaban dosing (*P* = 0.008 for both thrombus score and weight vs saline followed by 4F-PCC 300 IU/kg, Fig 4A and 4B). Some inhibition of thrombus formation was still apparent at 6 hours, but the difference was not significant (*P* = 0.17 for score, *P* = 0.21 for weight, Fig 4C); plasma levels of edoxaban were minimal at this time point. No thrombus formation was evident at 24 hours (Fig 4C and 4D). All significantly different comparisons had a statistical power of >90% given a sample size of 5.

#### Histopathological tissue analysis

No thrombus formation was observed in the kidney, liver, heart or brain. Thrombus formation was not found in the lung in animals receiving 4F-PCC (50 IU/kg). As expected based on previously generated data [19], a supratherapeutic dose of 4F-PCC (300 IU/kg) led to minimal lung thrombi formation (grade 1) in 3/5 animals at 30 minutes and 6 hours. After 24 hours, minimal thrombi (grade 1) were recorded in 3/5 animals, and moderate thrombus formation (grade 3) was recorded in 1/5 animals. The presence of edoxaban 600 μg/kg led to reductions in thrombus frequency across all time points investigated in animals receiving 4F-PCC 300 IU/kg: minimal thrombi (grade 1) recorded in 2/5 animals at 30 minutes, 6 hours and 24 hours post edoxaban dosing.

### Arterial thrombosis in rats: Comparison of 4F-PCC, aPCC and rFVIIa

**Fig 5:**
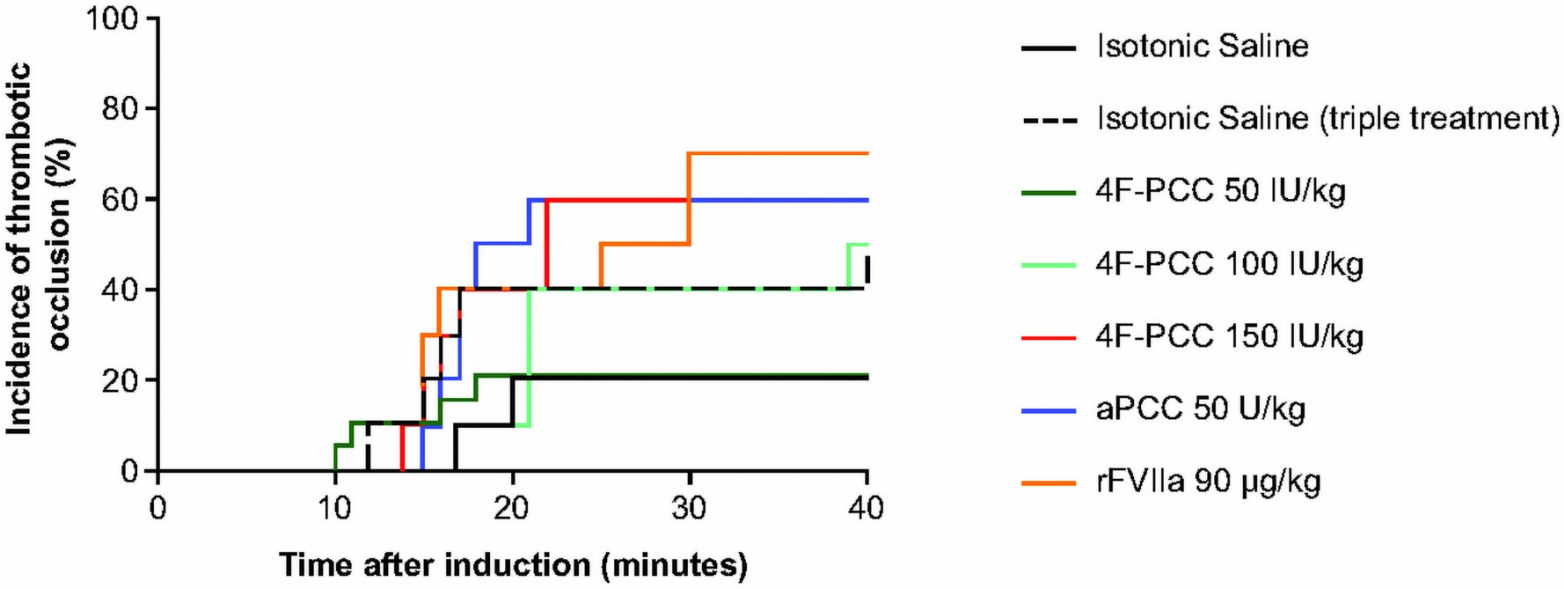
ATR assay: Comparison of 4F-PCC, aPCC and rFVIIa impact on thrombosis following FeCl_3_ treatment. Incidence of thrombosis after 5% FeCl_3_ injury and treatment with 4F-PCC, aPCC or rFVIIa.

4F-PCC demonstrated a dose-dependent increase in thrombosis incidence, with 50% and 70% of rats developing a thrombotic occlusion within 40 minutes post-dose of 4F-PCC 100 IU/kg and 150 IU/kg, respectively. At 40 minutes post-dose, the incidence of thrombotic occlusion was lower in rats administered 4F-PCC 50 IU/kg (21%) compared with rats administered aPCC 50 U/kg or rFVIIa 90 U/kg (60% in both groups) (Fig 5).

## Discussion

The thrombotic safety profile of PCC, aPCC and rFVIIa was assessed using three different animal models; two of these used a modified Wessler model of venous stasis-induced thrombosis in rabbits with dilutional coagulopathy or DOAC-induced coagulopathy, while the third model focused on arterial thrombosis in rats. All the models suggest that 4F-PCC has a low thrombotic potential compared with the other procoagulant agents tested. Our results suggest that prothrombotic risk with clinically relevant doses of 4F-PCC would be low compared with other procoagulant factor concentrates.

We did not see any thrombus formation with 4F-PCC up to the maximum therapeutic dose (50 IU/kg) in the rabbit model of venous stasis-induced thrombosis (including under conditions of haemodilution), while there was evidence of thrombus formation for aPCC and rFVIIa at doses as low as 10 IU/kg and 50 μg/kg, respectively. We observed thrombus formation with supratherapeutic doses of 4F-PCC (≥100 IU/kg); however, equivalent 4F-PCC levels are not expected to be reached in humans when dosing recommendations [33,34] are followed. When we studied the time course of thrombus formation at the supratherapeutic 4F-PCC dose of 300 IU/kg, we found that prothrombotic effects were apparent between 10 minutes and 6 hours following 4F-PCC infusion, but were not detectable thereafter; this demonstrates the transience of 4F-PCC effects on thrombus formation. However, it should be noted that the half-life of 4F-PCC is longer in humans [35] than in rabbits [32], which should be taken into account when considering the duration of potential thrombotic effects in patients.

Various clinical studies have investigated the thromboembolic safety of PCC in acquired coagulopathy (i.e. drug-induced coagulopathy, or coagulopathy occurring as the result of a systemic disorder). Reported risks of TEE are similar for both 4F-PCC and plasma in randomised controlled trials of VKA reversal [18,36–38], although real-world evidence from a retrospective patient chart review suggests that TEE risk may be greater for patients receiving 4F-PCC compared with FFP [39]. Based on retrospective studies and a systematic review, the TEE risk associated with clinical use of 4F-PCC in DOAC-mediated bleeding is low [40–44]. A review of PCC use in acquired coagulopathy noted a good safety profile in a cardiac surgery setting, with TEE incidence ranging from 0 to 6.2% [45], while studies of PCC use in trauma-induced coagulopathy show a TEE risk ranging up to 11.1% for PCC alone or in combination with FCH or FFP (no significant difference was observed compared with either product) [45]. Our results suggest that there is no increase in thrombotic risk when 4F-PCC is used in combination with FCH and/or TXA. Such data are reassuring, as these treatments may be used together in clinical practice [2,4]; however, it should be noted that the use of high-dose PCC (50 IU/kg) in combination with high-dose fibrinogen (100 mg/kg) was associated with increased risk of pulmonary emboli in a pig trauma model compared with PCC monotherapy [26]. Additionally, a risk of TEE has been demonstrated in the same model with high-dose (50 IU/kg) 4F-PCC [27]. 4F-PCC has also been associated with low TEE rates in liver disease-associated coagulopathy (ranging from 0 to 7%) [45]. Overall, TEE rates in cardiac surgery, trauma-induced coagulopathy and liver disease-associated coagulopathy appear to be in line with the background TEE incidence observed for these conditions [45], although there is no systematic data on the use of PCC under these different conditions.

Importantly, prothrombotic risk with a supratherapeutic dose of 4F-PCC (300 IU/kg) is not increased in the context of haemodilution. In haemodilution and in ‘true’ coagulopathy there may be concerns about a disproportionate reduction in coagulation inhibitor levels, which would in turn enhance the thrombogenicity of procoagulant agents. Our results indicate that this is not the case. In fact, thrombus scores were slightly lower in haemodiluted animals vs non-haemodiluted animals, suggesting that thrombotic risk may be offset to some extent by haemodilution. When assessing thrombotic risk associated with PCC administration following edoxaban treatment, no prothrombotic signal was seen with a clinically relevant dose of 4F-PCC (50 IU/kg). The data in the rabbit model of edoxaban-induced coagulopathy are supported by the rat model of FeCl_3_-induced arterial thrombosis: administration of 4F-PCC (50 IU/kg) was not associated with an increase in thrombus formation incidence versus saline in this setting, indicating that therapeutic doses of 4F-PCC do not increase prothrombotic risk. In contrast, therapeutic doses of aPCC and rFVIIa were both shown to increase the thrombus formation incidence versus saline, which is again in line with the results seen with the rabbit models and indicates that lower doses of aPCC or rFVIIa may be a treatment option in cases of trauma-induced coagulopathy.

It is important to amass data such as those we present here, to inform clinical use as local protocols may vary between trauma centres, and patient injuries are variable–using these model systems we are able to provide standardised evidence of potential risk, and to address the contributions of components of a treatment protocol (TXA, FCH) to overall risk. When speculating on the clinical relevance of our observations, it is important to be mindful of species-specific variations in coagulation, and in pharmacokinetics of PCC clearance [32,35]. One study in a pig model used to evaluate the safety of PCC in trauma suggests that pigs may become hypercoagulable in response to PCC, although this may vary according to dose used [27,46] and also according to co-treatment(s) [26]. ROTEM analysis of traumatised pigs (without PCC use) indicates the emergence of a hypercoagulable state in response to trauma [47], although there are a lack of corresponding data in untraumatised pigs, which hinders data interpretation. In addition, it should be considered that pigs have been reported to display a general tendency to hypercoagulability [48]. Species differences in coagulation profiles support a multi-species approach as there is no animal species available that is similar to humans in all relevant parameters [49]. Another potential limitation of the dilutional coagulopathy model used in the present study, on the other hand, is a lack of tissue injury and resulting tissue factor exposure. While the direct comparison of procoagulant treatments across animal models as applied in the present studies helps to derive conclusions on relative preclinical safety, for full understanding of procoagulant effects and the safety profiles of procoagulants, dedicated clinical trials would be necessary. It should also be noted that a limitation of this analysis is that it covers the results of three different studies, conducted over a number of years in different models, which may introduce some bias due to the different origins of the data.

In summary, these preclinical models of dilutional coagulopathy, edoxaban-induced coagulopathy and arterial thrombosis show a low thrombotic risk associated with 4F-PCC administration in rabbits and rats. 4F-PCC was not associated with thrombus formation at therapeutic doses in these models as opposed to results observed with aPCC or rFVIIa.

## Supporting information

S1 Fig 4F-PCC in combination with tranexamic acid or fibrinogen concentrate. (A) thrombus score; (B) thrombus weight. Data for venous stasis-induced thrombosis 10 minutes after the end of PCC infusion. 4F-PCC dose was 300 IU/kg, FCH dose was 100 mg/kg and TXA dose was 15 mg/kg. Figure shows mean (standard deviation).
(TIF)

S2 Fig 4F-PCC and fibrinogen concentrate following haemodilution. (A) thrombus score; (B) thrombus weight. Data for venous-stasis induced thrombosis 10 minutes after the end of 4F-PCC infusion. 4F-PCC dose was 300 IU/kg and FCH dose was 100 mg/kg. Figure shows mean (standard deviation).
(TIF)
