# Supplementary figures and images for: Evaluation of the prothrombotic potential of four-factor prothrombin complex concentrate (4F-PCC) in animal models
Source: PLoS One. 2021 Oct 6;16(10):e0258192. doi: 10.1371/journal.pone.0258192 (PMC8494364; doi:10.1371/journal.pone.0258192)

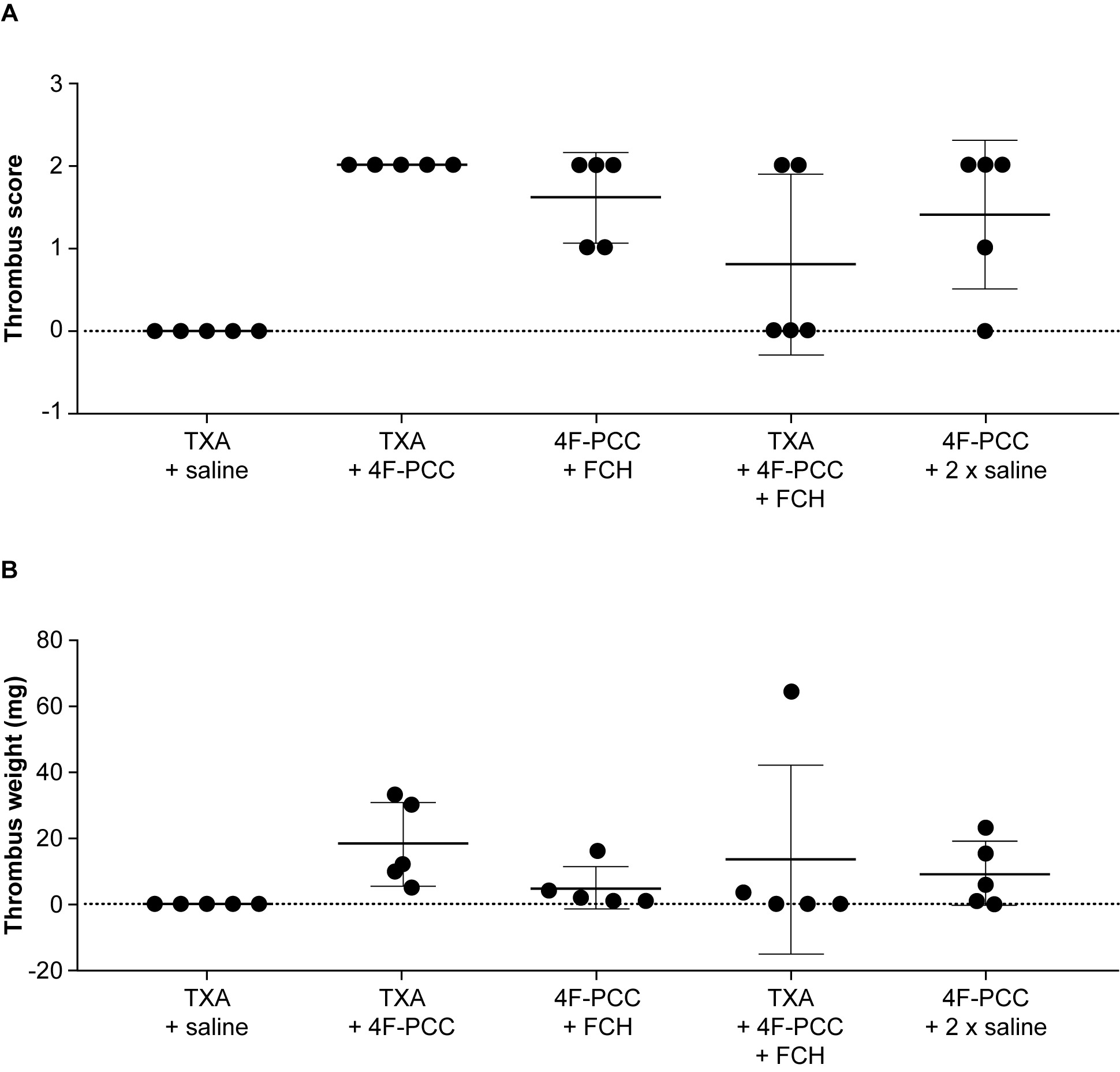

Supplement: S1 Fig — (A) thrombus score; (B) thrombus weight. Data for venous stasis-induced thrombosis 10 minutes after the end of PCC infusion. 4F-PCC dose was 300 IU/kg, FCH dose was 100 mg/kg and TXA dose was 15 mg/kg. Figure shows mean (standard deviation). (TIF) [file pone.0258192.s001.tif]

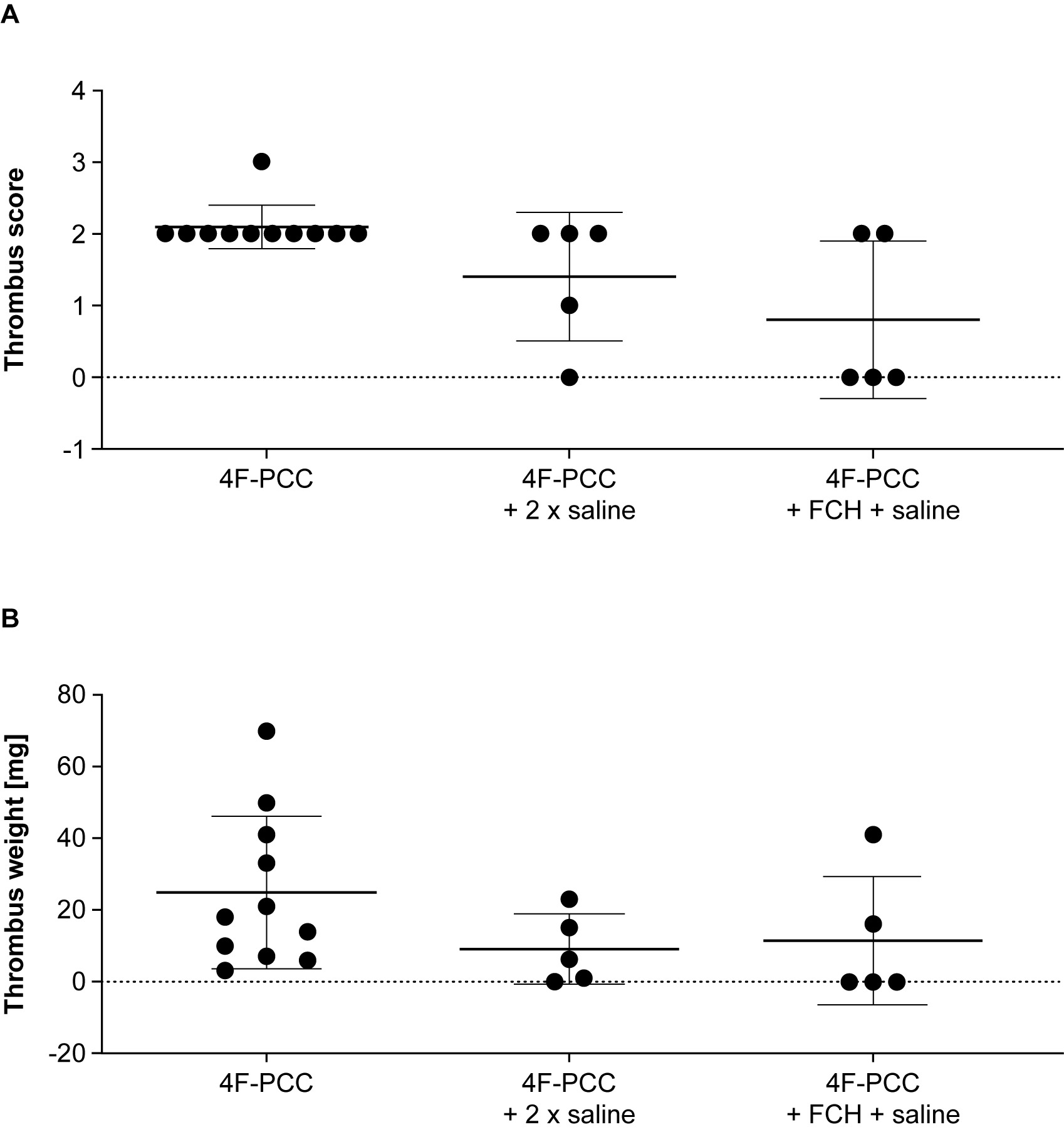

Supplement: S2 Fig — (A) thrombus score; (B) thrombus weight. Data for venous-stasis induced thrombosis 10 minutes after the end of 4F-PCC infusion. 4F-PCC dose was 300 IU/kg and FCH dose was 100 mg/kg. Figure shows mean (standard deviation). (TIF) [file pone.0258192.s002.tif]
